# Clinicopathological Features, Prognostic Factors and Survival in Patients With Pancreatic Cancer Bone Metastasis

**DOI:** 10.3389/fonc.2022.759403

**Published:** 2022-02-09

**Authors:** Ying Ren, Shicheng Wang, Bo Wu, Zhan Wang

**Affiliations:** ^1^ Department of Orthopedic Surgery, The Second Affiliated Hospital, Zhejiang University School of Medicine, Hangzhou, China; ^2^ Orthopedics Research Institute of Zhejiang University, Hangzhou, China; ^3^ Key Laboratory of Motor System Disease Research and Precision Therapy of Zhejiang Province, Hangzhou, China; ^4^ Department of Orthopedics, Ningbo No.6 Hospital, Ningbo, China; ^5^ Department of Orthopedic Oncology, Affiliated Hangzhou Cancer Hospital, Zhejiang University School of Medicine, Hangzhou, China

**Keywords:** pancreatic cancer, bone metastasis, prognosis, risk factor, clinicopathological features

## Abstract

**Purpose:**

The purpose of this study is to reveal the clinicopathological features and identify risk factors of prognosis among patients with pancreatic cancer bone metastasis (PCBM).

**Patients and Methods:**

Patients with PCBM were retrieved from the Surveillance, Epidemiology, and End Results (SEER) database between 2010 and 2016. Independent predictors for survival of those patients were determined by the univariate and multivariate Cox regression analysis. Forest plots were drawn by GraphPad 8.0.1 and used to visually display the results of multivariate analysis.

**Results:**

We identified 2072 eligible PCBM patients, of which 839 patients (40.5%) were female. Patients with age >60 years accounted for 70.6%. Multivariable Cox regression analysis indicated that age, pathological type, chemotherapy, liver metastasis, lung metastasis, and marital status were independent prognostic factors for both overall survival (OS) and cancer-specific survival (CSS). Kaplan–Meier survival curves showed that for patients with PCBM, age ≤60 years, non-ductal adenocarcinoma type, chemotherapy, no liver metastasis, no lung metastasis, and married status were correlated with increased survival. This population-based study showed that 1-year OS and CSS were 13.6% and 13.7%, respectively.

**Conclusion:**

The present study identified six independent predictors of prognosis in PCBM, including age, pathological type, chemotherapy, liver metastasis, lung metastasis, and marital status. Knowledge of these survival predictors is helpful for clinicians to accelerate clinical decision process and design personalized treatment for patients with PCBM.

## Introduction

Pancreatic cancer (PC) is a highly aggressive and metastatic malignancy, characterized by a high mortality. It has an extremely poor survival, with 5-year survival rate of less than 8% (1–3). Even if the diagnosis and treatment technology of PC improve, its prognosis has improved marginally over the past decades (4). The majority of PC patients develop metastasis either at the time of initial diagnosis or after initial diagnosis, which posts a new challenge for clinicians (3). He et al. (5) reported that liver and peritoneum metastases accounted for 45.1% and 49.9% of metastatic PC patients, respectively, which may be due to their anatomical sites (6). Other less common sites are the lung (11.4%), brain (0.4%), and bone (3.8%) (5). Although some previous studies focused on liver or lung metastasis of PC (7–9), studies on bone metastasis are scarce. The prevalence of bone metastasis in PC patients ranges from 5% to 20% (10, 11). Bone metastasis can result in skeletal related events (SREs), including hypercalcemia, bone pain, pathological fractures, spinal cord compression, and radiotherapy or surgery to the bone, which not only decreases the quality of life but also contributes to an unfavorable prognosis (12–14). Additionally, most bone metastases caused by pancreatic cancer are lytic lesions (15).

Previous studies reported that age, gender, tumor size, tumor location, histological type, T and N stages, histologic differentiation, radiotherapy, and chemotherapy were independent prognostic factors for PC (16, 17). Liu et al. (18) reported that age, gender, tumor size, alanine aminotransferase level (ALT) and CA19-9 were correlated with distant metastasis. Current therapeutic options for PC included surgical resection, radiotherapy, chemotherapy, immunotherapy, and et al. (19). However, there are few studies on the treatment of metastatic PC. To our knowledge, there are few studies reporting the pancreatic cancer bone metastasis (PCBM). Therefore, the present study was conducted to define the clinicopathological features and identify independent survival predictors of PCBM. To obtain insight into PCBM, we conducted a large-scale study by analyzing data of PCBM from the Surveillance, Epidemiology, and End Results (SEER) database.

## Materials and Methods

### Patient Selection

Data of patients diagnosed with PCBM, were extracted from the SEER*Stat version 8.3.9 software between 2010 and 2016. The SEER database collects malignant cancer information from 18 population-based cancer registries, representing approximately a third of the U.S. population (20). As the SEER database is public available and contains no patient-identified information, ethnic approval is not needed.

**Figure 1:**
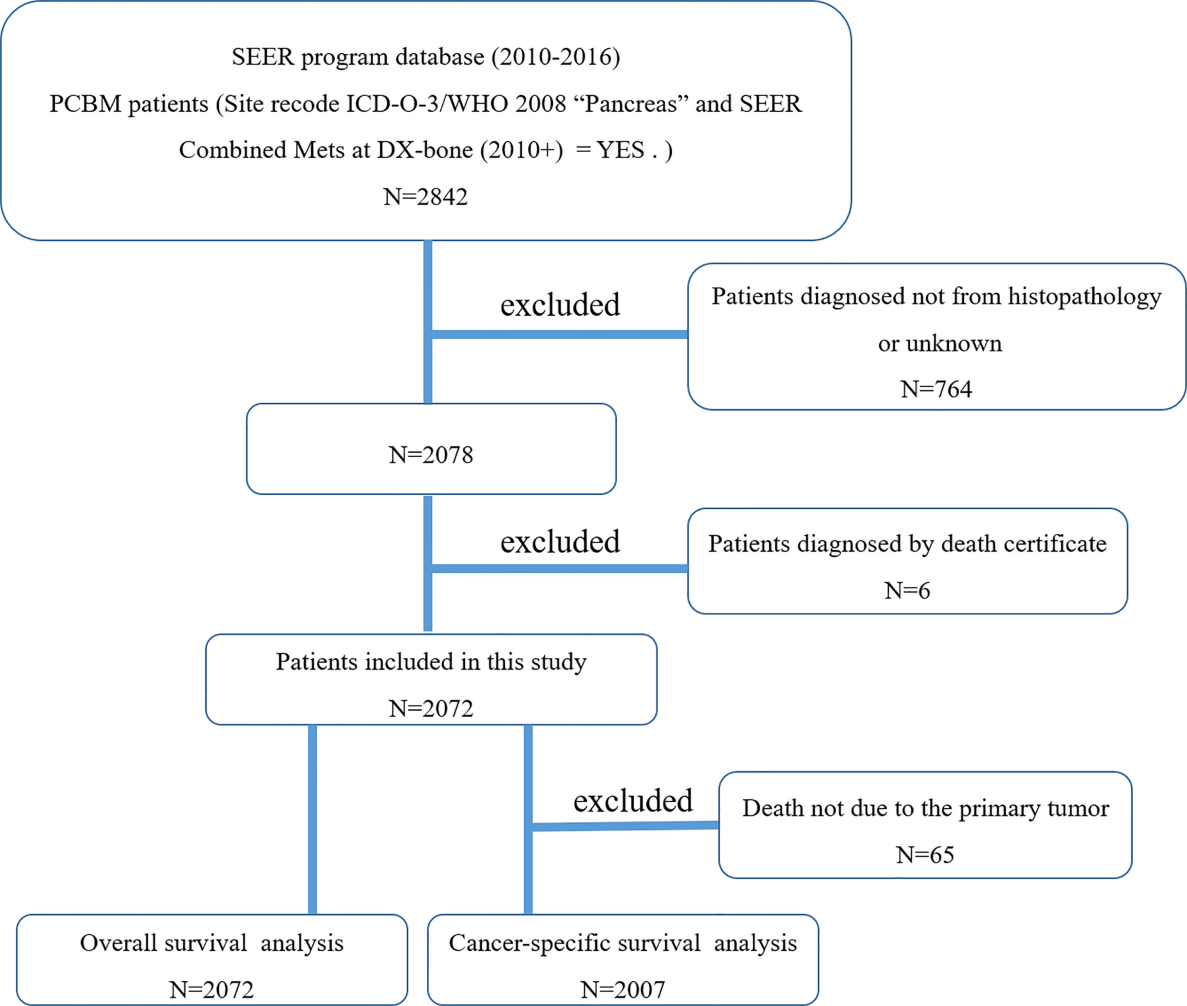
The flow chart for selection of study population. (SEER, Surveillance, Epidemiology, and End Results; ICD-O-3, international classification of diseases for oncology, 3^rd^ edition; PCBM, pancreatic cancer bone metastasis).

We first used the case-listing session to select the Site recode ICD-O-3/WHO 2008 “Pancreas”. Meanwhile, we set the SEER Combined Mets at DX-bone (2010+) to be YES. Thus, a total of 2842 patients with PCBM were enrolled. 764 patients diagnosed not by pathology were excluded. Additionally, six patients where the cancer was found upon autopsy or necropsy were excluded. **Figure 1** showed the flow chart for selection of study population. Variables obtained from the SEER database were race, gender, age at diagnosis, primary tumor site, pathological type, tumor size, surgery, radiotherapy, chemotherapy, organ metastases, marital status, vital status, survival time, and cause of death. Surgery or radiotherapy in the present study refers to treatment for primary tumor site. Overall survival (OS) was defined as the time from the date of diagnosis to the time of death from any cause (21, 22). Cancer-specific survival (CSS) was defined as the time from the date of diagnosis to the date of death from PC (21, 22).

### Statistical Methods

Univariate and multivariable Cox regression models were used to identify independent predictors of OS and CSS. Meanwhile, hazard ratio (HR) and their 95% confidence interval (95% CIs) were presented in both univariate and multivariate analysis. Kaplan–Meier survival curves were applied to compare the differences among these groups by the log-rank test. Chi-square test is used to compare the rates of two groups. SPSS 21.0 software was used to conduct all above statistical tests and risk factors of *p*<0.05 were considered as significant predictors. Additionally, we drew Forest plots by GraphPad 8.0.1 to visually display the results of multivariate analysis.

## Results

### Clinicopathologic Characteristics

We identified 2072 eligible patients with PCBM. The baseline characteristics of all patients in the current study were summarized in the **Table 1**. Among the population, 79.5%, 11.4%, and 9.1% of patients were white, black, and other races, respectively. More than half of the patients (n=1233, 59.5%) were males. As for age, more patients with age > 60  years (n=1463, 70.6%) were observed. There were 518(25.0%), 285(13.8%), and 503(24.3%) of cases located in head of pancreas, body of pancreas, and tail of pancreas, respectively. Ductal adenocarcinoma accounted for 75.1% of all pathological type. 899(43.4%) of patients presented with tumor size <5 cm, and 511(24.7%) of patients presented with tumor size ≥ 5 cm. Regarding the treatment, more than half of the patients (51.9%) received chemotherapy, while only 24.7% and 1.4% of patients received radiotherapy and surgery, respectively. As for visceral metastasis, only 3.3% of patients presented with brain metastasis, while 28.7% and 38.8% of patients developed liver and lung metastases, respectively. In addition, more than half of the patients (n=1233, 59.5%) got married. The 1-year OS and CSS rates of the population were 13.6% and 13.7%, respectively.

**Table 1:** Characteristics of 2072 patients with pancreatic cancer bone metastasis.

Variable	Value
**Race**	
White	1647 (79.5%)
Black	237 (11.4%)
Others	188 (9.1%)
**Gender**	
Female	839 (40.5%)
Male	1233 (59.5%)
**Age (years)**	
≤60	609 (29.4%)
>60	1463 (70.6%)
Mean	66
Median	67
**Primary site**	
Head of pancreas	518 (25.0%)
Body of pancreas	285 (13.8%)
Tail of pancreas	503 (24.3%)
Others*	766 (37.0%)
**Pathological type**	
Ductal adenocarcinoma	1557 (75.1%)
Non-ductal adenocarcinoma	515 (24.9%)
**Tumor size (cm)**	
<5	899 (43.4%)
≥5	511 (24.7%)
Unknown	662 (31.9%)
**Surgery**	
Yes	28 (1.4%)
No	2044 (98.6%)
**Radiotherapy**	
Yes	512 (24.7%)
No	1560 (75.3%)
**Chemotherapy**	
Yes	1075 (51.9%)
No	997 (48.1%)
**Brain metastasis**	
No	1906 (92.0%)
Yes	69 (3.3%)
Unknown	97 (4.7%)
**Liver metastasis**	
No	1445 (69.7%)
Yes	594 (28.7%)
Unknown	33 (1.6%)
**Lung metastasis**	
No	1266 (61.1%)
Yes	804 (38.8%)
Unknown	2 (0.1%)
**Marital status**	
Married	1137 (54.9%)
Others	843 (40.7%)
Unknown	92 (4.4%)
**Dead**	
Yes	1874 (90.4%)
No	198 (9.6%)
**1-year OS rate**	13.60%
**1-year CSS rate**	13.70%

OS, overall survival; CSS, cancer-specific survival. *Others: C25.3-Pancreatic duct, and C25.4-Islets of Langerhans, C25.7-Other specified parts of pancreas, C25.8-Overlapping lesion of pancreas, C25.9-Pancreas, NOS.

### Univariate Cox Regression Analysis

Potential risk factors for the OS and CSS by univariate analysis are summarized in **Table 2**. The results showed that age (>60 years vs. ≤60 years, HR=1.265; 95% CI, 1.143-1.399; *p*<0.001), primary site (Body of pancreas vs. Head of pancreas, HR=0.918, 95% CI, 0.787-1.071, *p*=0.277; Tail of pancreas vs. Head of pancreas, HR=1.078, 95% CI, 0.947-1.226, *p*=0.254; Others vs. Head of pancreas, HR=1.159, 95% CI, 1.031-1.302, *p*=0.014), pathological type (Non-ductal adenocarcinoma vs. Ductal adenocarcinoma, HR=0.689; 95% CI, 0.618-0.769; *p*<0.001), local radiotherapy (No vs. Yes, HR=1.236; 95% CI, 1.112-1.374; p<0.001), systemic chemotherapy (No vs. Yes, HR=2.334; 95% CI, 2.125-2.565; *p*<0.001), liver metastasis (Yes vs. No, HR=1.301; 95% CI, 1.176-1.440; *p*<0.001), lung metastasis (Yes vs. No, HR=1.288; 95% CI, 1.174-1.414; *p*<0.001), and marital status(Others vs. Married, HR=1.195; 95% CI, 1.088-1.313; *p*<0.001), were significantly associated with OS. In terms of CSS, age (>60 years vs. ≤60 years, HR=1.264; 95% CI, 1.141-1.400; *p*<0.001), primary site (Body of pancreas vs. Head of pancreas, HR=0.912, 95% CI, 0.779-1.068, *p*=0.254; Tail of pancreas vs. Head of pancreas, HR=1.086, 95% CI, 0.952-1.238, *p*=0.219; Others vs. Head of pancreas, HR=1.157, 95% CI, 1.027-1.304, *p*=0.016), pathological type (Non-ductal adenocarcinoma vs. Ductal adenocarcinoma, HR=0.681; 95% CI, 0.609-0.762; *p*<0.001), local radiotherapy (No vs. Yes, HR=1.228; 95% CI, 1.103-1.366; p<0.001), systemic chemotherapy (No vs. Yes, HR=2.367; 95% CI, 2.150-2.606; *p*<0.001), liver metastasis (Yes vs. No, HR=1.293; 95% CI, 1.166-1.434; *p*<0.001), lung metastasis (Yes vs. No, HR=1.288; 95% CI, 1.171-1.416; *p*<0.001), and marital status(Others vs. Married, HR=1.181; 95% CI, 1.073-1.299; *p*<0.001), were significant predictors.

**Table 2:** Univariate Cox analysis of variables in pancreatic cancer bone metastasis.

Variable	OS		CSS	
	HR (95% CI)	*p*	HR (95% CI)	*p*
**Race**				
White	1		1	
Black	1.005 (0.870-1.160)	0.948	0.982 (0.847-1.139)	0.808
Others	1.007 (0.858-1.183)	0.929	1.007 (0.844-1.168)	0.932
**Gender**				
Female	1		1	
Male	1.075 (0.980-1.179)	0.124	1.071 (0.975-1.177)	0.152
**Age (years)**				
≤60	1		1	
>60	1.265 (1.143-1.399)	<0.001	1.264 (1.141-1.400)	<0.001
**Primary site**				
Head of pancreas	1		1	
Body of pancreas	0.918 (0.787-1.071)	0.277	0.912 (0.779-1.068)	0.254
Tail of pancreas	1.078 (0.947-1.226)	0.254	1.086 (0.952-1.238)	0.219
Others	1.159 (1.031-1.302)	0.014	1.157 (1.027-1.304)	0.016
**Pathological type**				
Ductal adenocarcinoma	1		1	
Non-ductal adenocarcinoma	0.689 (0.618-0.769)	<0.001	0.681 (0.609-0.762)	<0.001
**Tumor size (cm)**				
<5	1		1	
≥5	1.087 (0.969-1.220)	0.156	1.082 (0.962-1.216)	0.188
**Radiotherapy**				
Yes	1		1	
No	1.236 (1.112-1.374)	<0.001	1.228 (1.103-1.366)	<0.001
**Chemotherapy**				
Yes	1		1	
No	2.334 (2.125-2.565)	<0.001	2.367 (2.150-2.606)	<0.001
**Brain metastasis**				
No	1		1	
Yes	1.064 (0.827-1.368)	0.629	1.117 (0.863-1.445)	0.4
**Liver metastasis**				
No	1		1	
Yes	1.301 (1.176-1.440)	<0.001	1.293 (1.166-1.434)	<0.001
**Lung metastasis**				
No	1		1	
Yes	1.288 (1.174-1.414)	<0.001	1.288 (1.171-1.416)	<0.001
**Marital status**				
Married	1		1	
Others	1.195 (1.088-1.313)	<0.001	1.181 (1.073-1.299)	<0.001

### Multivariate Cox Regression Analysis

**Figure 2:**
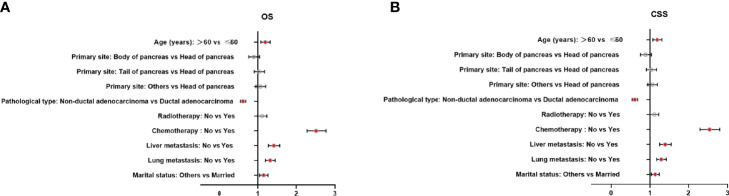
Forest plots of the predictors of OS **(A)** and CSS **(B)** in patients with PCBM. (PCBM, pancreatic cancer bone metastasis; OS, overall survival; CSS, cancer-specific survival).

**Figure 3:**
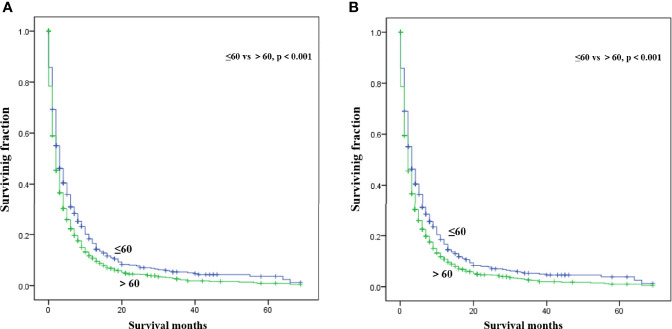
Kaplan-Meier survival curves for estimating OS **(A)** and CSS **(B)** in patients with PCBM based on age.

**Figure 4:**
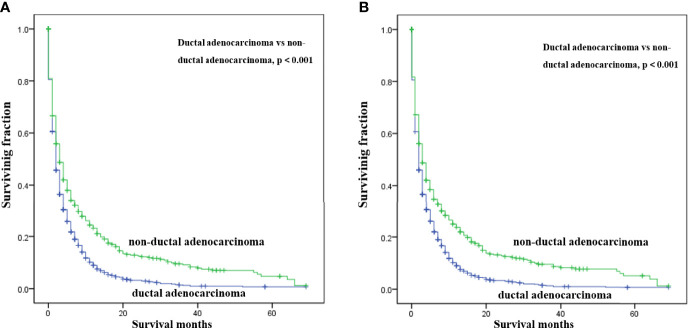
Kaplan-Meier survival curves for estimating OS **(A)** and CSS **(B)** in patients with PCBM based on pathological type.

**Figure 5:**
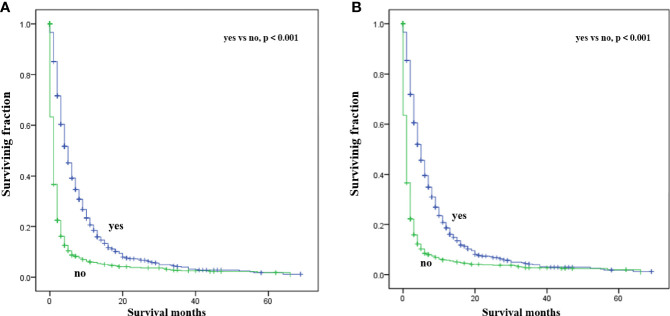
Kaplan-Meier survival curves for estimating OS **(A)** and CSS **(B)** in patients with PCBM based on chemotherapy.

**Figure 6:**
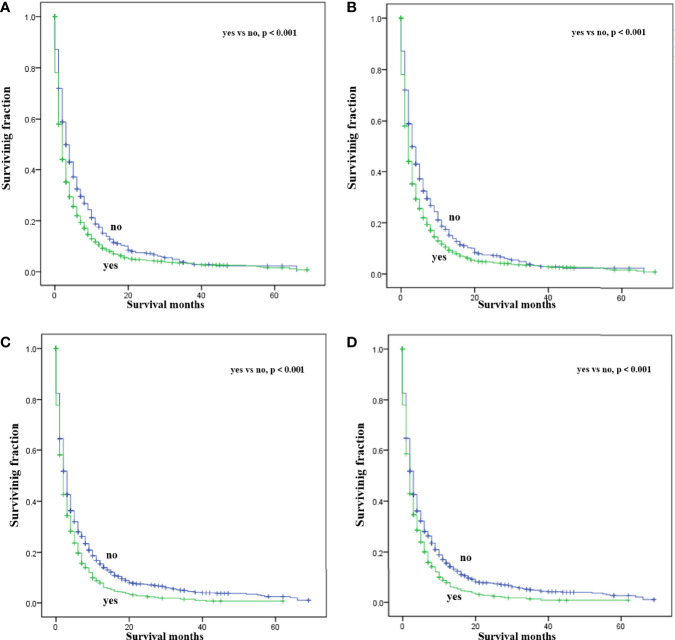
Kaplan-Meier survival curves for estimating OS and CSS in patients with PCBM based on liver **(A, B)** and lung **(C, D)** metastasis.

**Figure 7:**
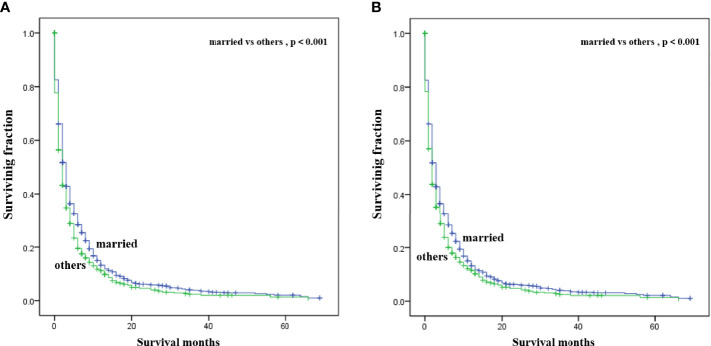
Kaplan-Meier survival curves for estimating OS **(A)** and CSS **(B)** in patients with PCBM based on marital status.

Multivariate analysis of the OS and CSS among patients with PCBM presented in **Table 3** and **Figure 2**. Multivariable Cox regression analysis indicated that age (>60 years vs. ≤60 years, HR=1.193; 95% CI, 1.077-1.321; *p*<0.001), pathological type (Non-ductal adenocarcinoma vs. Ductal adenocarcinoma, HR=0.615; 95% CI, 0.549-0.688; *p*<0.001), systemic chemotherapy (No vs. Yes, HR=2.512; 95% CI, 2.277-2.771; *p*<0.001), liver metastasis (Yes vs. No, HR=1.414; 95% CI, 1.275-1.568; *p*<0.001), lung metastasis (Yes vs. No, HR=1.318; 95% CI, 1.198-1.450; *p*<0.001), and marital status(Others vs. Married, HR=1.150; 95% CI, 1.046-1.265; *p*=0.004) were independent prognostic factors for OS. In terms of CSS, age (>60 years vs. ≤60 years, HR=1.190; 95% CI, 1.072-1.320; *p*<0.001), pathological type (Non-ductal adenocarcinoma vs. Ductal adenocarcinoma, HR=0.606; 95% CI, 0.539-0.681; *p*<0.001), systemic chemotherapy (No vs. Yes, HR=2.537; 95% CI, 2.295-2.804; *p*<0.001), liver metastasis (Yes vs. No, HR=1.401; 95% CI, 1.261-1.556; *p*<0.001), lung metastasis (Yes vs. No, HR=1.302; 95% CI, 1.181-1.434; *p*<0.001), and marital status(Others vs. Married, HR=1.134; 95% CI, 1.030-1.250; *p*=0.011) were independent risk factors. Additionally, we drew Kaplan–Meier survival curves to visually show these independent prognostic factors. Patients with age ≤ 60 years were significantly associated with better outcomes (**Figure 3**). Patients with ductal adenocarcinoma type exhibited worse survival outcomes (**Figure 4**). Chemotherapy had a positive effect on prolonging the life of patients (**Figure 5**). Liver and lung metastases were associated with poor survival in patients with PCBM (**Figure 6**). Notably, longer OS and CSS were observed in married patients (**Figure 7**). The survival curves of patients in different groups decreased with time. In the first 20 months, the survival curve of patients in each group dropped rapidly.

**Table 3:** Multivariate Cox analysis of variables in pancreatic carcinoma bone metastasis.

Variable	OS		CSS	
	HR (95% CI)	*p*	HR (95% CI)	*p*
**Age (years)**				
≤60	1		1	
>60	1.193 (1.077-1.321)	0.001	1.190 (1.072-1.320)	0.001
**Primary site**				
Head of pancreas	1		1	
Body of pancreas	0.893 (0.765-1.043)	0.153	0.881 (0.752-1.033)	0.118
Tail of pancreas	1.029 (0.902-1.173)	0.673	1.032 (0.903-1.179)	0.647
Others	1.069 (0.949-1.205)	0.273	1.063 (0.942-1.201)	0.322
**Pathological type**				
Ductal adenocarcinoma	1		1	
Non-ductal adenocarcinoma	0.615 (0.549-0.688)	<0.001	0.606 (0.539-0.681)	<0.001
**Radiotherapy**				
Yes	1		1	
No	1.109 (0.997-1.234)	0.057	1.110 (0.996-1.237)	0.059
**Chemotherapy**				
Yes	1		1	
No	2.512 (2.277-2.771)	<0.001	2.537 (2.295-2.804)	<0.001
**Liver metastasis**				
No	1		1	
Yes	1.414 (1.275-1.568)	<0.001	1.401 (1.261-1.556)	<0.001
**Lung metastasis**				
No	1		1	
Yes	1.318 (1.198-1.450)	<0.001	1.302 (1.181-1.434)	<0.001
**Marital status**				
Married	1		1	
Others	1.150 (1.046-1.265)	0.004	1.134 (1.030-1.250)	0.011

## Discussion

Although treating PC has made remarkable progress over the past century, the prognosis remains poor mainly due to difficulties in its early diagnosis and metastasis (23). Bone metastasis represents an underappreciated site of metastasis among PC patients. As indicated in the present study, patients with PCBM experienced quite poor survival with 1-year OS and CSS rates of less than 15%. However, factors influencing their survival remain poorly understood. Previous studies of metastatic PC have been limited by small sample sizes, lack of population-based data. To our knowledge, the present study is the first population-based analysis to explore the survival predictors for patients with PCBM.

In this observational study, age at diagnosis was proved to be an independent survival predictor for patients with PCBM, which was consistent with previous findings among all PC patients (24–26). Patients over 60 years old are generally considered the elderly. Based on previous literature (27, 28), we divided the patients’ age into >60 years old and ≤60 years old for convenient analysis. Although some researchers reported tumor site was an independent survival predictor of PC (29–31), our multivariate Cox regression analysis showed that tumor site was not correlated with OS or CSS in patients with PCBM. For convenient analysis, patients’ tumor size were divided into<5 and ≥5 cm based on previous literature (21, 28). However, univariate Cox regression analysis showed tumor size was not correlated with survival. Pancreatic ductal adenocarcinomas (PDACs) account for approximately 95% of all PC pancreatic cancers (32). In our study, ductal adenocarcinoma subtype (75%) also represented most histological subtype of patients with PCBM. Moreover, ductal adenocarcinoma subtype was an important independent predictor for worse CSS and OS of patients with PCBM. Maybe non-ductal tumors have unique morphology and biology that is distinct from that of ductal neoplasms of the pancreas (33). Further researches are needed to clarify its specific mechanism. Lung or liver metastasis worsens the prognosis of patients with PCBM, which were in line with previous studies on PC (34, 35). Interestingly, better prognosis was observed in married patients, which may be associated with family social support. Similar results were supported by other studies on PC patients (36, 37). In **Supplement Table 1**, we found that among married patients, the proportions of white patients, male patients, patients with age>60 years, patients undergoing surgery and chemotherapy were higher.

The impact of chemotherapy to increase survival of PCBM remains unknown. This retrospective data analysis using a large cancer database suggests that use of chemotherapy could improve survival in patients with PCBM. Additionally, chemotherapy can stabilize health-related quality of life and improve pain control among advanced PC patients (38, 39). As for radiotherapy, our study did not find its survival benefits for patients with PCBM. Surgery is the mainstay of treatment for non-metastatic PC patients (40). Due to the small number of patients with PCBM receiving surgery in the current study, we did not analyze the its effect on survival. Additionally, previous studies indicated that surgical resection of oligometastatic disease after PDAC might have benefit for prolonging survival (41–43). Thus, research efforts should focus on exploring the role of surgery in patients with PCBM in the future. In summary, this study provides support for the selection of clinical treatment for patients with PCBM.

Although the SEER database provides a large amount of clinical data, there are still many limitations in our research. First, we need to further conduct a follow-up clinical trial to verify this result. Second, the role of surgery on prognosis were not analyzed due to the small number of cases who received surgery. Finally, the performance status, lymph node metastasis, and CEA or CA19-9 levels, were not available in the SEER database, which need to be further studied.

## Conclusion

Our population-based study showed that age ≤60, non-ductal adenocarcinoma type, chemotherapy, no liver metastasis, no lung metastasis, and married status were independent predictors for better OS and CSS, which may have implications for future clinical practice. However, further studies are warranted to validate these results.

## Data Availability Statement

The raw data supporting the conclusions of this article will be made available by the authors, without undue reservation.

## Ethics Statement

Ethical review and approval was not required for the study on human participants in accordance with the local legislation and institutional requirements. Written informed consent for participation was not required for this study in accordance with the national legislation and the institutional requirements.

## Author Contributions

ZW conceived and designed the study. YR, SW, and BW collected the data. YR and SW performed the statistical analysis. YR wrote the manuscript. ZW and BW revised it. All authors read and approved the final manuscript.

## Funding

This work was supported by the China Postdoctoral Science Foundation (2021M692792), National Natural Science Foundation of China (82103499), and Zhejiang Provincial Natural Science Foundation (LQ22H160040).

## Conflict of Interest

The authors declare that the research was conducted in the absence of any commercial or financial relationships that could be construed as a potential conflict of interest.

## Publisher’s Note

All claims expressed in this article are solely those of the authors and do not necessarily represent those of their affiliated organizations, or those of the publisher, the editors and the reviewers. Any product that may be evaluated in this article, or claim that may be made by its manufacturer, is not guaranteed or endorsed by the publisher.
