# Supplementary material for: Clinicopathological Features, Prognostic Factors and Survival in Patients With Pancreatic Cancer Bone Metastasis
Source: Front Oncol. 2022 Feb 9;12:759403. doi: 10.3389/fonc.2022.759403 (PMC8863857; doi:10.3389/fonc.2022.759403)
Supplement: Supplementary file 1 [file Table_1.docx]

| **Supplement Table 1. Association between married status and clinicopathological features** | | | |
| --- | --- | --- | --- |
|  | **Married （n=1137）** | **Others (n=843)** | ***p*** |
| **Race** |  |  | ＜0.001 |
| White | 943 | 635 |  |
| Black | 78 | 148 |  |
| Others | 116 | 60 |  |
| **Gender** |  |  | ＜0.001 |
| Female | 366 | 436 |  |
| Male | 771 | 407 |  |
| **Age (years)** |  |  | ＜0.001 |
| ≤60 | 295 | 286 |  |
| ＞60 | 842 | 557 |  |
| **Primary site** |  |  | 0.085 |
| Head of pancreas | 295 | 206 |  |
| Body of pancreas | 158 | 117 |  |
| Tail of pancreas | 295 | 188 |  |
| Others | 389 | 332 |  |
| **Pathological type** |  |  | 0.473 |
| Ductal adenocarcinoma | 863 | 628 |  |
| Non-ductal adenocarcinoma | 274 | 215 |  |
| **Tumor size (cm)** |  |  | 0.468 |
| ＜5 | 509 | 366 |  |
| ≥5 | 285 | 201 |  |
| Unknown | 343 | 276 |  |
| **Surgery** |  |  | 0.008 |
| Yes | 23 | 5 |  |
| No | 1114 | 838 |  |
| **Radiotherapy** |  |  | 0.782 |
| Yes | 284 | 206 |  |
| No | 853 | 637 |  |
| **Chemotherapy** |  |  | ＜0.001 |
| Yes | 655 | 376 |  |
| No | 482 | 467 |  |
| **Brain metastasis** |  |  | 0.606 |
| No | 1054 | 774 |  |
| Yes | 37 | 27 |  |
| Unknown | 46 | 42 |  |
| **Liver metastasis** |  |  | 0.389 |
| No | 312 | 253 |  |
| Yes | 806 | 579 |  |
| Unknown | 19 | 11 |  |
| **Lung metastasis** |  |  | 0.875 |
| No | 700 | 512 |  |
| Yes | 436 | 330 |  |
| Unknown | 1 | 1 |  |
